# Supplementary material for: Learning curve and surgical outcome of robotic assisted colorectal surgery with ERAS program
Source: Sci Rep. 2022 Nov 29;12:20566. doi: 10.1038/s41598-022-24665-w (PMC9709162; doi:10.1038/s41598-022-24665-w)
Supplement: Supplementary file 1 — Supplementary Information 1. [file 41598_2022_24665_MOESM1_ESM.zip › LSC Raw data-Ñ[▒K/073/P_ñw╝╨░O▒Kñσ.pdf]

[redacted]

Pathologic diagnosis:
1. Rectum and sigmoid colon, lapa anterior resection --- Adenocarcinoma, moderately differentiated. Tumor invaded to pericolorectal soft tissue and metastasized to mesocolic lymph nodes (4/27). The bilateral cut ends are free of tumor.
2. IMA root, dissection --- Unremarkable fibrovascular tissue and lymph node (0/1).
3. Colon, cut end, distal, resection --- Chronic inflammation.
4. "Lymph node", IMA, dissection --- Unremarkable fibroadipose tissue. No lymph node seen.

Ancillary study for diagnosis:
IHC stains for MLH1, MSH2, PMS2 and MSH6 are done on section D. All above markers are preserved.

Prognostic and predictive factor:
1. Tumor size: 4.2x3.5 cm.
2. Depth of invasion: Tumor invades to pericolorectal tissue.
3. Histologic grade: G2: Moderately differentiated.
4. Angiolymphatic invasion: Not identified.
5. Perineural invasion: Not identified.
6. Tumor deposits (discontinuous extramural extension): Not identified.
7. Macroscopic tumor perforation: Not identified.
8. Surgical Margins: a) Proximal Margin: Uninvolved by invasive carcinoma, distance of tumor from margin: 5.7 cm. b) Distal Margin: Uninvolved by invasive carcinoma, distance of tumor from margin: 3.4 cm. c) Radial or Mesenteric Margin: Uninvolved by invasive carcinoma, distance of tumor from margin: 3 cm.
9. Lymph node status: Involved (4/28).
10. Extranodal involvement: Not identified.
11. Tumor regression grading S/P CCRT: No known presurgical therapy.
12. Type of polyp in which invasive carcinoma arose: Not identified.
13. Additional pathologic findings: Not identified.
14. Pathological TNM Stage: pT3N2a(According to the eighth edition, American Joint Committee on Cancer Staging Guidelines for Tumors).
15. TNM descriptors: Not applicable.

Gross description:
The specimen consists of 1) a segment of colon, 13 cm in length and 3.5 cm in proximal cut end circumference, 3.5 cm in distal circumference, and mesocolic soft tissue. A gray tan firm annular elevated and centrally ulcerative tumor, 4.2x3.5 cm, is noted, 3.4 cm to the nearest cut end. On cross section, the tumor invades to the mesocolic soft tissue with 30 mm to the radial margin. The both cut ends are free of tumor grossly. 2) IMA root, a piece of tan yellow soft tissue, 3.7x2.8x1 cm. 3) distal cut end, a piece of tan mucosa-covered soft tissue, 1.8x1.5x0.8 cm. 4) IMA LN, a piece, 1x0.7x0.3 cm. Representative part for section: A) proximal cut end B) distal cut end C-E) tumor F) colon G) radial margin H-K) specimen 2-4 L-P) lymph nodes.

Note:
1. The interpretation of MMR panel and recommended studies are as below: (1) No loss of nuclear expression of MMR proteins: low probability of microsatellite instability-high (MSI-H) . (2) Please note: There are exceptions to the above IHC interpretations. These results should not be considered in isolation, and clinical correlation with genetic counseling is recommended to assess the need for germline testing.
2. This case has been peer reviewed by two doctors.
#T-59300_2 #M-81403_2 2 1105 S0000F
#T-C4560_2 #M-81406_2 2 1105 000000

已電子簽章

敬告：本單僅供醫師參考使用，不具任何申請效力！！
請病患妥為保管，若不慎遺失需自負個人資料外洩責任！
